# MicroPro: using metagenomic unmapped reads to provide insights into human microbiota and disease associations

**DOI:** 10.1186/s13059-019-1773-5

**Published:** 2019-08-06

**Authors:** Zifan Zhu, Jie Ren, Sonia Michail, Fengzhu Sun

**Affiliations:** 1 Quantitative and Computational Biology Program, Department of Biological Sciences, University of Southern California, Los Angeles, CA USA; 2 Department of Pediatrics, Division of Gastroenterology, Keck School of Medicine, University of Southern California, Los Angeles, CA USA

**Keywords:** Metagenomics, Next-generation shotgun sequencing, Human disease, Microbiome, Virus

## Abstract

We develop a metagenomic data analysis pipeline, MicroPro, that takes into account all reads from known and unknown microbial organisms and associates viruses with complex diseases. We utilize MicroPro to analyze four metagenomic datasets relating to colorectal cancer, type 2 diabetes, and liver cirrhosis and show that including reads from unknown organisms significantly increases the prediction accuracy of the disease status for three of the four datasets. We identify new microbial organisms associated with these diseases and show viruses play important prediction roles in colorectal cancer and liver cirrhosis, but not in type 2 diabetes. MicroPro is freely available at https://github.com/zifanzhu/MicroPro.

**Electronic supplementary material:**

The online version of this article (10.1186/s13059-019-1773-5) contains supplementary material, which is available to authorized users.

## Introduction

Trillions of microbes populate various sites of the human body and form microbiome communities [1]. These microorganisms and their interactions between each other and the host play an important role in many physiological processes including metabolism, reproduction and immune system activity [2, 3]. In the nineteenth century, culture-based methods demonstrated that changes in these microbes might lead to disease. Since then, many subsequent studies confirmed these findings [4]. However, the cultivation technology only provided a limited view since many microorganisms could not be cultured in vitro [5]. Over the past 20 years, and thanks to the rapid development of sequencing technology, sequencing-based methods have gradually replaced the cultivation technology and have become the most widely used tools for microbial analysis. The 16S ribosomal RNA sequencing together with the recent shotgun whole genome sequencing not only discovers large amounts of non-cultivable microbes, but also fundamentally changes the way microbial analysis is performed [6, 7]. Researchers are now finding more evidence correlating human microbiota with various diseases such as colorectal cancer [8], type 2 diabetes [9, 10], liver cirrhosis [11], and many others. In addition, human microbiota has been linked to the effectiveness of cancer chemotherapy [12]. In some studies, a single species or strain is associated with a disease while in other cases, groups of microorganisms interact to affect human health [13].

Mounting evidence connecting the microbiome with disease description has gradually brought about the concept of a supervised predictive study of microorganisms for different diseases. Although most of the studies are merely observational, which means we cannot simply conclude the causality between microbes and the disease [7], the existing correlations are sufficient to prove that performing a predictive study about the effect of microbiota on diseases is plausible. More specifically, many advances in this area have made it possible to predict the existence or states of a certain disease given information of the microorganisms for a specific subject.

In the field of machine learning, a supervised predictive study aims to build models based on sets of features to maximally approximate the response value or correctly classify the label of a sample. In the microbiota-disease setting, the response can either be disease/non-disease or different subtypes within a disease; thus, a classification version of supervised predictive study is desired [14]. However, the selection of features varies greatly among different studies. Our study is focused on analyzing the microbial abundance in the context of shotgun whole genome sequencing. A similar analysis can also be applied to other choices of the feature including operational taxonomic units (OTUs, widely used in 16S rRNA analysis) [15], NCBI non-redundant Clusters of Orthologous Groups (COG) [16], or Kyoto Encyclopedia of Genes and Genomes (KEGG) groups [17]. With many software packages like MetaPhlAn2 [18] or Centrifuge [19] tackling the computation of the microorganisms’ abundance, the microbiota-disease predictive study can be formulated as a machine learning task based on a species-by-sample matrix with qualitative labels.

Recently, many studies have focused on the predictive analysis between human microbiota and diseases. For example, Zeller et al. [8] developed a species abundance-based LASSO [20] model to differentiate between colorectal cancer patients and healthy individuals. Qin et al. [11] used gene markers to predict liver cirrhosis based on a Support Vector Machine (SVM) [21]. Moreover, Pasolli et al. [22] built a database named curatedMetagenomicData, which stored uniformly processed microbiome analysis results across 5716 publicly available shotgun metagenomic samples. Using this database, Pasolli et al. developed a random forest [23] model to analyze the predictive power of different microbial features (such as species abundance, pathway coverage) on various diseases.

However, the currently available approaches face a few challenges. First, in microbiome studies, there are generally two types of methods for microbial abundance characterization from metagenomic datasets: reference-based methods and de novo assembly-based methods. Many reference-based methods involve the process of mapping short reads against known microbial reference sequences in the NCBI RefSeq database [24] or a catalog of taxon-associated marker sequences [18]. Microbial abundances can be estimated from the mapping results. However, a large proportion of the reads cannot be successfully mapped to a particular reference, which results in the potential loss of valuable information. On the other hand, de novo assembly-based methods do not need any reference genomes or marker sequences. These methods create metagenomic assembled groups (MAGs) by first assembling the reads into contigs, then binning the metagenomic contigs into MAGs, and finally estimating the abundance profiles of the MAGs. For example, Xing et al. [25] and Ren et al. [26] both identified microbial species in the metagenomic datasets through de novo assembling reads into contigs and then binning contigs into MAGs and analyzed disease association with the relative abundance of the MAGs. De novo assembly-based methods have the potential to capture microbes without reference genomes, thus solving the main problem of the reference-based methods. However, de novo assembly-based methods also have their own issues. Sequence assembly is computationally expensive and takes a lot of time and memory. For example, Minia 3 [27] took 53 h and 63 GB memory to perform de novo assembly while reference-based method, Centrifuge [19], completed the mapping in less than 2 h and used 4 GB memory on the same machine for the QinJ_T2D dataset.

Secondly, the roles of viruses in diseases are often neglected. Within the human microbial community, bacterium reads constitute the majority while virus reads are reported as a small proportion of the total reads (less than 5% in datasets analyzed in our study). Additionally, an incomplete database of viral reference genomes and the high mutation rates of viruses make them even more challenging to characterize and analyze [28]. Therefore, most disease-related microbiome studies focus only on the connection between bacteria and the disease. However, learning about viruses is important as the number of viruses is about 10 times that of bacteria [29], and they can play important roles in multiple diseases. Norman et al. [30] showed that enteric virome change happened in patients with inflammatory bowel disease and bacteriophages might serve as antigens in the human immune system. Ren et al. [26] demonstrated that decreased viral diversity was observed in patients with liver cirrhosis as compared to healthy individuals. Reyes et al. [31] identified disease-discriminatory viruses associated with childhood malnutrition, which might help to characterize gut microbiota development. Therefore, the role of viruses in human diseases should be investigated.

In order to overcome the challenges mentioned above, we developed a metagenomic predictive pipeline, MicroPro, which analyzes data in three main steps: (1) reference-based known microbial abundance characterization—perform taxonomic profiling based on sequence alignment against reference genomes; (2) assembly-binning-based unknown organism feature extraction—use cross-assembly to assemble the combined unmapped reads from all samples and consider each assembled contig as originated from an “unknown” organism, which refers to an organism with no known references available in the database. Since some contigs may originate from the same organism, we cluster assembled contigs into bins and then treat each bin as an “unknown” organism; and (3) machine learning predictive analysis—apply machine learning tools for predicting disease/non-disease or disease states based on the species-by-sample matrix. To the best of my knowledge, this is the first predictive pipeline based on a combination of both known and unknown microbial organisms. We tested MicroPro on four public NGS datasets and showed that consideration of unknown organisms significantly increased the prediction accuracy for three of the four datasets. Furthermore, we systematically investigated the effect of viruses on multiple diseases with the virus version of MicroPro. We examined the predictive power of the model with known and unknown viruses and showed that unknown viruses played an important role in disease prediction warranting further attention.

## Results

### MicroPro: a metagenomic disease-related prediction analysis pipeline taking unmapped reads into consideration

**Fig. 1:**
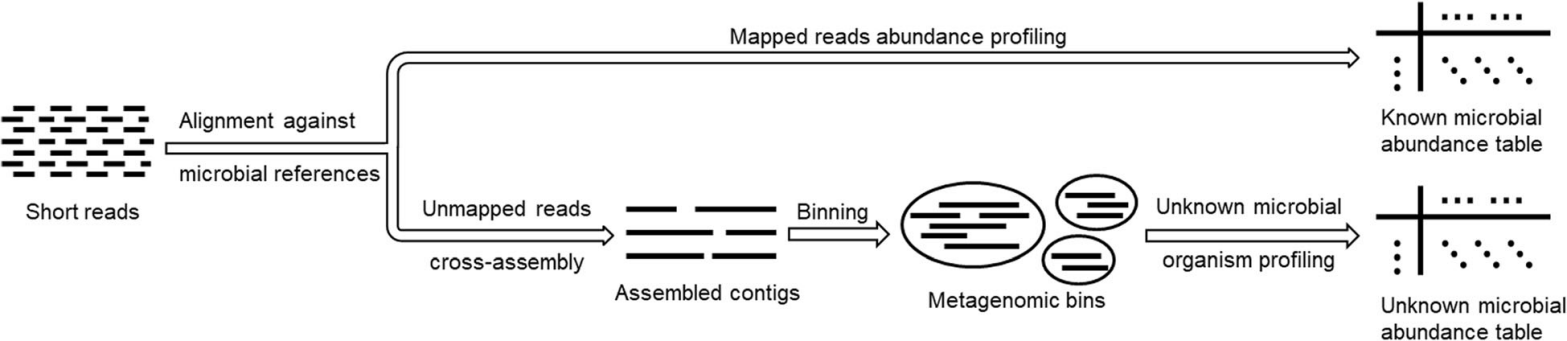
Procedures of microbial abundance characterization in MicroPro

We developed a new metagenomic analysis pipeline, MicroPro, to take into account both known and unknown microbial organisms for the prediction of disease status. MicroPro consists of three main steps: (1) reference-based known microbial abundance characterization, (2) assembly-binning-based unknown organism feature extraction, and (3) machine learning predictive analysis. Figure 1 presents the procedures to extract the abundance table of both known and unknown microbial organisms. Various machine learning tools can then be applied to study the association between microbial abundances and the disease. Detailed explanations of each step are available in the “Methods” section.

### Comparison between MicroPro, reference-based method, and de novo assembly-based method on simulated dataset

**Fig. 2:**
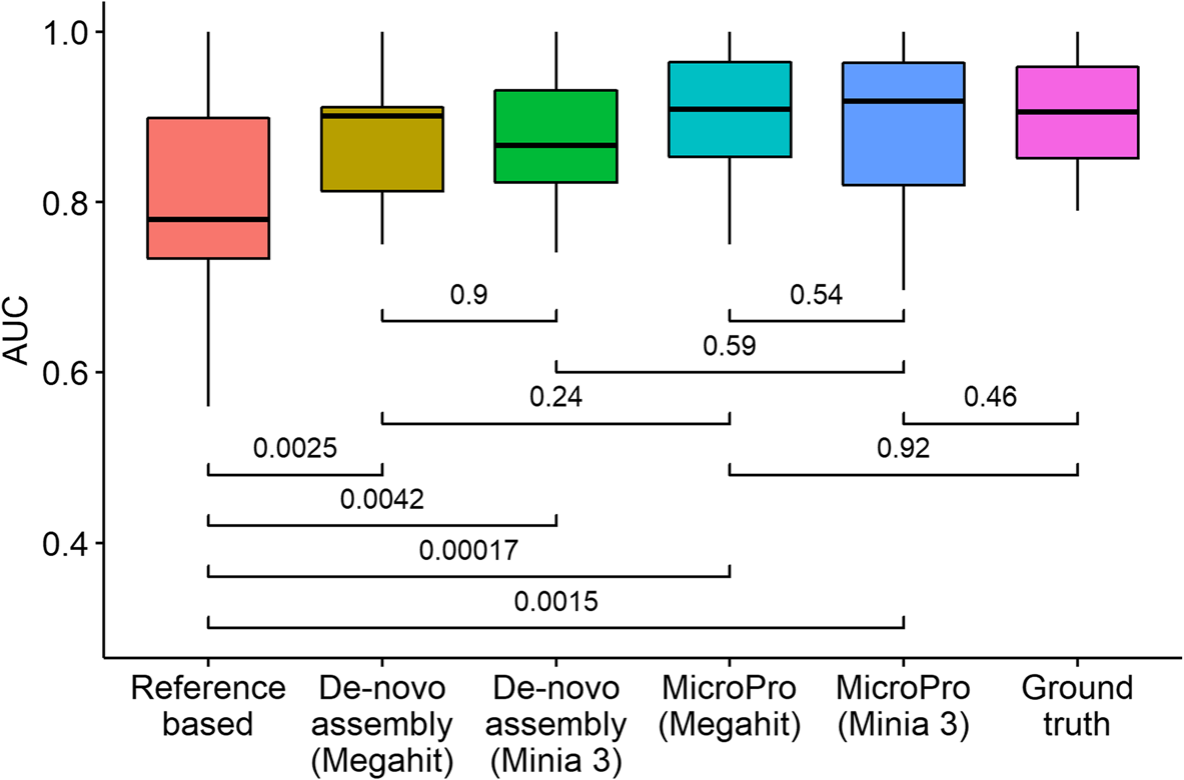
Results of simulation studies. Boxplots of random forest AUC scores obtained using features from different methods are provided. Each random forest classification model was repeatedly trained and tested 30 times. Student’s *t* test *p* values between pairs of methods are given

We simulated 50 metagenomic shotgun sequenced samples (25 cases and 25 controls) consisting of bacteria from 100 genera. Each sample had a size of 1 GB (500 Mbp). The details of the simulation setup are described in the “Methods” section. We then tested MicroPro and compared it with the reference-based method and the de novo assembly-based method on the simulated dataset for their prediction performance of disease status. The reference-based method only used the known microbial abundances produced in the first step of MicroPro to perform the classification study. On the other hand, the de novo assembly-based method skipped the first step of MicroPro and performed assembly and binning on the whole dataset. The simulation study showed that the predictive performance of the reference-based method was significantly lower than that of the de novo assembly-based method and MicroPro, since reference-based method only captured microbes within the reference database which possibly ignored other microbes important for the classification. De novo assembly-based method and MicroPro had a similar performance in terms of prediction, as they both used all the reads in the sample without the information loss encountered in the reference-based method (Fig. 2). However, in terms of computational cost, the reference-based method needed the fewest computing resources as sequence alignment was computationally cheaper than assembly. Additionally, de novo assembly-based method required at least twice the wall time and 1.5 times the memory compared to MicroPro. This result was not unexpected since sequence assembly was the computational bottleneck for these two methods and MicroPro only assembled unmapped reads while de novo assembly-based method assembled all of them (Table 1). In summary, MicroPro performed better in prediction than reference-based method and required much fewer computing resources than de novo assembly-based method.

**Table 1:** Wall time and memory use for individual methods applied to the simulated dataset

	Wall time (min)	MaxRSS (GB)
Reference-based	14	0.2
MicroPro (Megahit)	81	12
MicroPro (Minia 3)	43	6
De novo assembly-based (Megahit)	305	20
De novo assembly-based (Minia 3)	79	9

Computing resources required for the individual methods used in the simulation study are provided. MaxRSS refers to maximum memory used by the corresponding method

Sczyrba et al. [32] showed that Megahit [33] and Minia 3 [27] were among the top assemblers and produced contigs of similar quality in the Critical Assessment of Metagenome Interpretation (CAMI) challenge. To compare these two assemblers, we tested Megahit and Minia 3 in the simulation study and found that they had a similar performance in prediction (Fig. 2), but Minia 3 was computationally more efficient than Megahit (Table 1).

### Application of MicroPro to four real metagenomic datasets

We downloaded four publicly available shotgun-sequenced metagenomic datasets related to three different diseases: colorectal cancer (CRC) [8], type 2 diabetes (T2D) [9, 10], and liver cirrhosis (LC) [11] (Table 2).

**Table 2:** Four large-scaled metagenomic datasets spanning three different diseases

Dataset name	Disease	Sample size	Number of cases	Number of controls	Data size (Gbp)	Reference
Zeller_CRC	CRC	184	91	93	915	Zeller et al. [8]
Karlsson_T2D	T2D	96	53	43	296	Karlsson et al. [9]
QinJ_T2D	T2D	145	71	74	376	Qin et al. [10]
QinN_LC	LC	237	123	114	1200	Qin et al. [11]

Detailed information of the four metagenomic datasets analyzed in this paper is provided

**Fig. 3:**
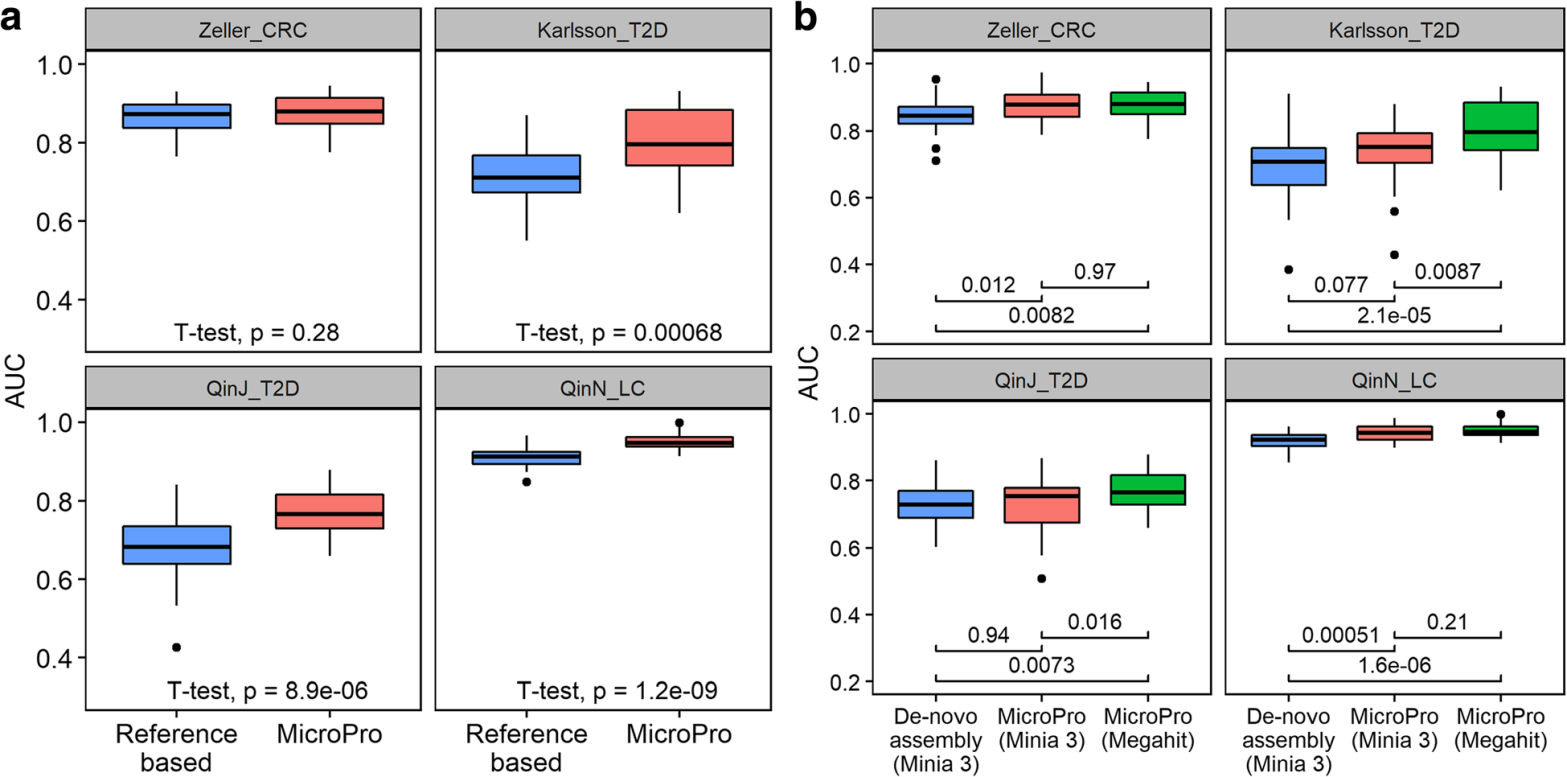
Prediction results on four real metagenomic datasets. **a** Boxplots of random forest AUC scores obtained by reference-based method and MicroPro (with assembler Megahit). Each random forest classification model was repeatedly trained and tested 30 times. Student’s *t* test *p* values are given. **b** Boxplots of random forest AUC scores obtained by MicroPro and de novo assembly-based method. Results of MicroPro with two different assemblers are shown. Each random forest classification model was repeatedly trained and tested 30 times. Student’s *t* test *p* values between pairs of methods are given

We then analyzed these four datasets using MicroPro. We found that MicroPro significantly improved the prediction accuracy over reference-based method in three of the four datasets (Karlsson_T2D, QinJ_T2D, and QinN_LC). This result uncovered the predictive value of the abundance profiles of unknown organisms that were commonly ignored by many reference-based metagenomic analysis pipelines (Fig. 3a). We also compared MicroPro with de novo assembly-based method. Due to insufficient computing memory, we only used Minia 3 for de novo assembly. The prediction results showed that MicroPro (Minia 3) performed slightly better than de novo assembly-based method with the AUC increase being significant in Zeller_CRC and QinN_LC and weakly significant in Karlsson_T2D (Fig. 3b). As in the simulation study, the de novo assembly-based method was computationally more expensive than MicroPro (Additional file 2: Table S1). Moreover, we compared the performance of MicroPro using two different assemblers: Megahit and Minia 3. The results showed that MicroPro (Megahit) performed significantly better than MicroPro (Minia 3) in datasets Karlsson_T2D and QinJ_T2D and both had a similar prediction accuracy in the other two datasets (Fig. 3b). Again, Megahit required much more computing resources than Minia 3 (Additional file 2: Table S1). It suggests that for small datasets or with ample computing resources, Megahit is a better choice over Minia 3 for real data. Unless specified, all the following analyses are based on Megahit-assembled contigs.

### Analysis of the role of unknown viruses in virus-only prediction study

**Fig. 4:**
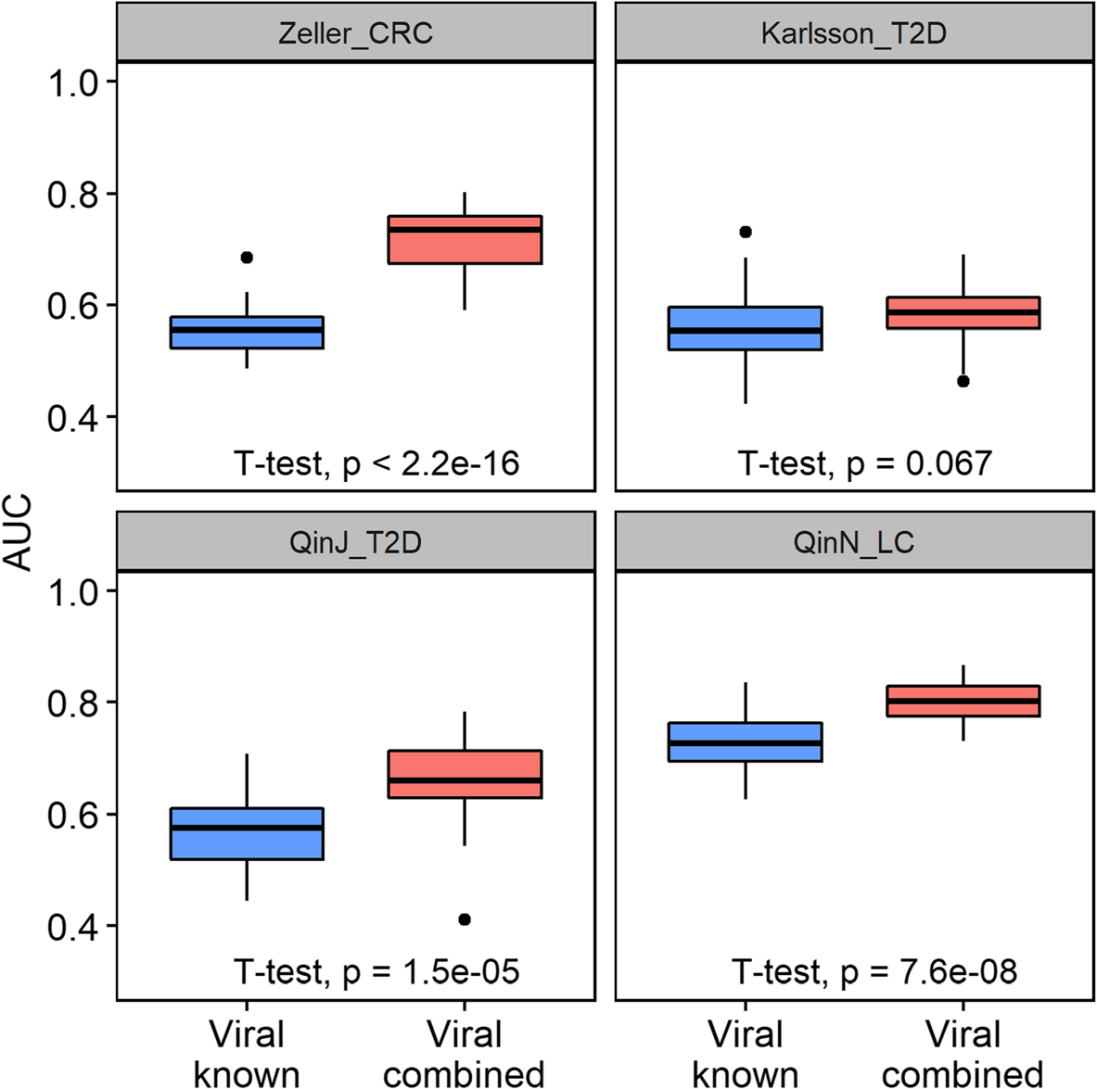
Prediction results on four real metagenomic datasets only using viral abundances. Boxplots of random forest AUC scores obtained using different viral features are provided. “Viral known” refers to only using known viral abundances to perform the classification while “Viral combined” means using both known and unknown viral abundances. Each random forest classification model was repeatedly trained and tested 30 times. Student’s *t* test *p* values are given

To test the predictive power of the viral organisms within the microbial community, we applied the virus version of MicroPro to all the four datasets. Although the prediction accuracy obtained by the abundance profiles of known viruses was much lower than that obtained by known microbial abundances including bacteria, adding the unknown feature significantly improved the prediction accuracy for datasets Zeller_CRC, QinJ_T2D, and QinN_LC (Fig. 4). For Zeller_CRC and QinJ_T2D, the role of unknown viruses was remarkable as they increased the average AUC score from 0.55 to 0.72 and 0.56 to 0.65, respectively. For QinN_LC, the average AUC score with known viruses was 0.73 which was much better than the other three datasets, and the inclusion of unknown viral abundances further increased it to 0.80. These results highlight the advantage of MicroPro to consider both known and unknown microbial organisms in metagenomic prediction study and further demonstrate the important association of viruses, especially unknown viruses with multiple diseases.

On the other hand, we acknowledge that the increase in prediction accuracy for Karlsson_T2D is weaker than the other three datasets. Considering the fact that there were only 28 unknown viral contigs found for this dataset (Additional file 3: Table S2), the number of unknown viruses were too small to play a major role in the prediction analysis hence the low AUC increment. However, in the other T2D dataset QinJ_T2D, much more viral contigs were discovered (Additional file 3: Table S2), suggesting that the detection of viral contigs can be dataset-dependent with confounding factors like sample collection method and shotgun sequencing protocols affecting the generated metagenomic reads. For prediction performance using both known and unknown viruses, QinN_LC (mean AUC = 0.80) and Zeller_CRC (mean AUC = 0.72) are much higher than Karlsson_T2D (mean AUC = 0.58) and QinJ_T2D (mean AUC = 0.65), which indicates the potential weaker prediction role of viruses in T2D compared to the other two diseases.

### Alpha diversity analysis of the abundance profiles of both microbial organisms and viruses

**Fig. 5:**
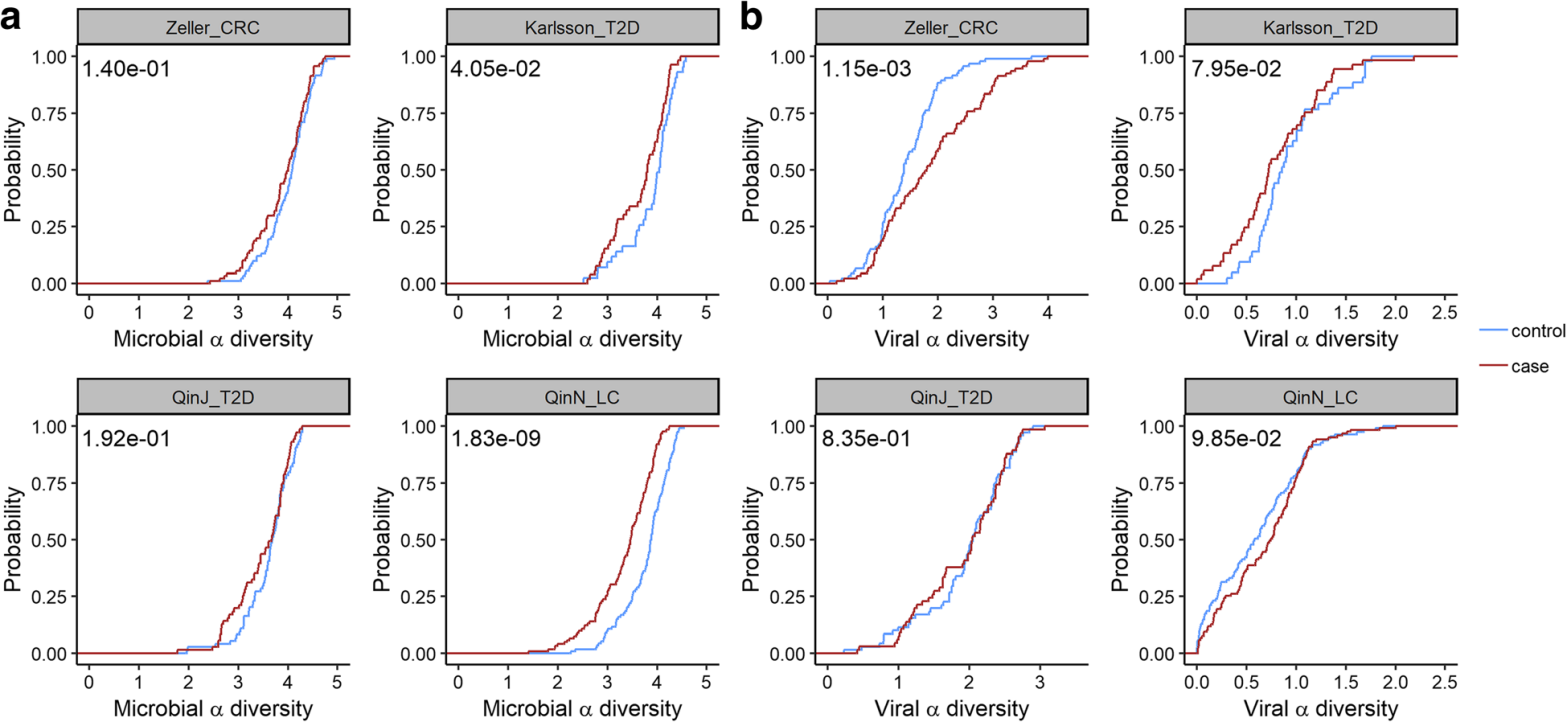
Cumulative probability of the alpha diversity. Cumulative probability distributions of alpha diversity with Shannon index are shown. Abundance profiles of both known and unknown organisms are used for the calculation. Plot **a** uses the abundance profiles of all the microbes while plot **b** only uses the abundance profiles of viruses. *p* values based on the WMW test for the alpha diversity between the cases and the controls are provided

We also performed alpha diversity analysis for both microbial and viral abundance profiles in the cases and controls. Figure 5 shows the results of using the abundance profiles of both known and unknown microbial organisms. Alpha diversity results based on the abundance profiles of only known or unknown organisms are provided in Additional file 1: Figure S1-S2. For microbial alpha diversity (Fig. 5a), a consistent pattern of the case being less diverse is observed. This pattern is most remarkable for QinN_LC, which corresponds to its high AUC score when using microbial abundances to differentiate between cases and controls (Fig. 3a). For the viral alpha diversity, we did not identify statistically significant differences between cases and controls for liver cirrhosis (QinN_LC) and type 2 diabetes (Karlsson_T2D, QinJ_T2D) at the type I error of 0.05. Surprisingly, we discovered that the viral diversity in CRC cases is much higher than that in the healthy controls, a finding consistent with the result from a recent study of Nakatsu et al. [34] that analyzed the viromes in CRC cases and controls.

### Significantly associated microbial organisms for each disease

We explored the microbial organisms that were significantly associated with a certain disease in the metagenomic analysis. In our study, significantly associated microbial organisms were selected by the Boruta feature selection method [35]. Table 3 illustrates that a majority of the selected microbes are unknown, further highlighting the advantage of our pipeline to characterize unknown microbes from unmapped reads. Detailed information about the selected microbes in each dataset, including mean abundances in cases and controls, is provided in Additional file 4: Table S3. We further discussed the novel microbe-disease associations discovered in this study (see the “Discussion” section). These discoveries can lay groundwork for future mechanistic understanding of the pathophysiology of the corresponding diseases.

**Table 3:** Summary of significantly associated microbes for each dataset

	# Significant microbes	# Known	# Unknown
Zeller_CRC	49 (2313)	8 (1287)	41 (1026)
Karlsson_T2D	25 (1379)	4 (785)	21 (594)
QinJ_T2D	21 (1411)	5 (925)	16 (486)
QinN_LC	68 (1442)	21 (936)	47 (506)

Numbers of significantly associated microbes for each dataset are provided. “# Significant microbes”, “# Known” and “# Unknown” represent the number of selected significant, known and unknown microbes, respectively. Numbers shown in the parenthesis are the corresponding total count of microbes

### Taxonomic assignments of the MAGs generated in four datasets

To further identify the taxonomic assignment of the MAGs derived in each dataset, we calculated the pairwise distance between each MAG and the reference genomes in the Centrifuge database (up to December 10, 2018) with Mash v.2.0 [36], a widely used alignment-free genome comparison tool based on the overlap of *k*mers between genomes. We found that none of the pairwise Mash distance was below 0.05, a threshold suggested by the authors for distinguishing microbial genomes at the species level [36], which showed that the MAGs generated in all the four datasets did not overlap with the genomes in the Centrifuge database at the species level. Nayfach et al. [37] suggested Mash distance of 0.35 as a genus-level threshold for microbes. Using this threshold, we found that 5.8–10.3% of the MAGs for the four datasets could be classified to the genus level (Additional file 5: Table S4).

### Prediction analysis between two T2D datasets

**Fig. 6:**
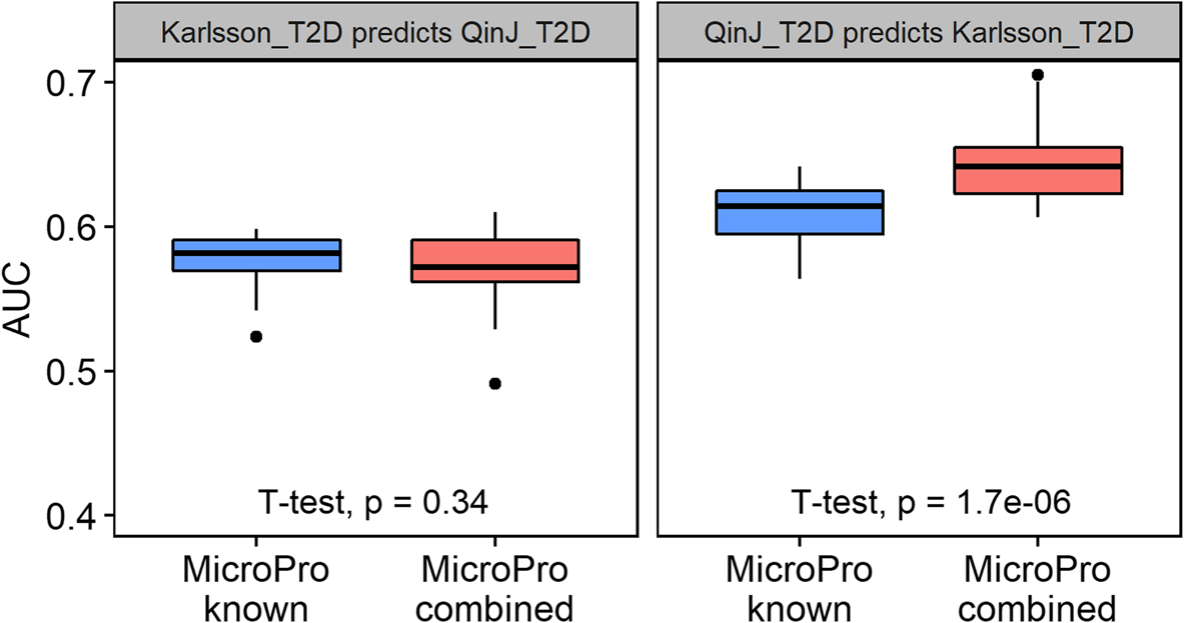
Prediction analysis between two T2D datasets. Boxplots of random forest AUC scores obtained in the cross-study analysis are provided. “MicroPro known” refers to using only known microbial abundance profile extracted by MicroPro as the feature while “MicroPro combined” refers to using both known and unknown abundances. Each random forest classification model was repeatedly trained and tested 30 times. Student’s *t* test *p* values are given

Although prediction within one study can give good results, prediction accuracy drops sharply when applied to a different dataset. Different experiment protocols, various sequencing platforms, and variable time points of data collection are all possible reasons for the drop in the prediction accuracy. In our study, there were two T2D datasets, which offered an opportunity to analyze the generalization potential of the predictive model across different studies. As shown in Fig. 6, the AUC scores dropped markedly for both cases from above 0.75 to around 0.6 when compared with the prediction within one study (Fig. 3a). When using Karlsson_T2D to predict QinJ_T2D, adding the unknown feature seemed to have no effect on the prediction accuracy. However, in the other case, adding the unknown features significantly increased the AUC scores suggesting that in cross-study settings, adding unknown organisms can result in higher prediction accuracy.

## Discussion

Many studies have described the development of computational tools to investigate the association of microbial organisms with complex traits. However, most of the available reference-based tools focus on the microbial species with a known reference genome, and the reads not mapped to the known genomes are not considered, which can result in the loss of potentially useful information. Other de novo assembly-based methods demand significant computing resources with long computational time and large memory requirement. In order to address these issues, we developed the MicroPro pipeline that extracts both known and unknown microbial features within metagenomic datasets. We tested MicroPro in a disease prediction study involving four public metagenomic datasets covering three different diseases. We show that the prediction accuracy is significantly increased when adding unknown microbial features for three of the four datasets, which demonstrates the important predictive role of unknown organisms. Additionally, since MicroPro only assembles the unmapped reads, it is computationally much more efficient than de novo assembly-based methods.

Many studies have demonstrated the important role of viruses in human diseases like inflammatory bowel disease [30] and liver cirrhosis [26]. However, due to the limited virus genome database and high mutation rates, viruses were often neglected in metagenomic association studies. The virus version of MicroPro aims at extracting both known and unknown viral features from sequenced reads. We performed prediction analysis with viral abundances extracted by the virus version of MicroPro on the same public metagenomic datasets. The results indicated that viruses did play some roles in diseases like colorectal cancer and liver cirrhosis. Thus, the role of viruses should not be ignored in the metagenomic analysis. Also, for some datasets, like Zeller_CRC in our study, the power of predicting disease when using known virus only was close to random guess. However, the inclusion of unknown viral features remarkably increased the prediction accuracy. This demonstrated that our pipeline was able to distinguish the role of viruses by investigating unknown features.

We also discovered many novel microbial associations with specific diseases and disease prediction. Some of these associations are consistent with what has been described in the past. We discovered a number of organisms which were predictive of liver cirrhosis. These organisms include *Veillonella parvula*, *Veillonella rodentium*, *Fusobacterium periodonticum*, *Lactobacillus salivarius*, and *Selenomonas* sp. *oral taxon 136*. These organisms frequently inhabit the oral cavity, and many are pathogenic. For example, *Veillonella parvula* is a bacterium in the genus *Veillonella*. *Veillonella* are Gram-negative bacteria anaerobic cocci. *Veillonella parvula* is well known for its lactate fermenting abilities and inhabit the intestines and oral mucosa. In humans, *Veillonella* can cause osteomyelitis, endocarditis, periodontitis, and dental caries as well as various systemic infections [38]. Similarly, *Fusobacterium* is a genus of anaerobic, Gram-negative, non-spore-forming bacteria, similar to *Bacteroides*. Although in the past, *Fusobacterium* was considered part of the normal oral microbiome, the current consensus is that *Fusobacterium* should always be treated as a pathogen [39] and has been linked to periodontal diseases, ulcerative colitis, and colon cancer. These organisms originate from the mouth but may also inhabit the intestine [40]. Even though our model discovered new organism associations for disease prediction, it has been shown that the oral microbiota can influence the gut microbiome and has been detected in the stools of patients with cirrhosis [11]. Chen et al. [41] described *Veillonella* and other oral microbiota as discriminative taxa between patients with cirrhosis compared to controls. The permissive oral microbial invasion may be related to altered hepatic bile production or the frequent use of proton pump inhibitors in this population. Both bile and gastric acid are natural gates that can inhibit the survival of many of the ingested organisms. Furthermore, bacterial populations originating from the oral microbiota are capable of producing high levels of methyl mercaptan (CH3SH). Elevated blood levels of CH3SH have been linked to the development of hepatic encephalopathy [42]. The presence of both *Dialister pneumosintes* and *Parvimonas micra* was predictive of the development of colorectal cancer in our model. *Dialister pneumosintes* was found in patients with periodontitis [43] and has been shown to have potential pathogenic roles in various human body sites including the lung and brain [44]. It has been recently shown to be an important component of the dysbiotic microbiome in patients with gastric cancer [45]. *Parvimonas micra* can cause infectious endocarditis [46], native joint septic arthritis [47], and spondylodiscitis [48] and has also been associated with gastric cancer [45]. Not only enrichment of specific organism was predictive of colorectal cancer in our model, but we also report depletion of specific organisms, such as *Cutibacterium acnes*, is seen in association with this type of cancer. While this organism was originally described in subjects with acne, it can still be found throughout the digestive tract [49] and was originally named *Propionibacterium acnes* for its ability to generate propionic acid [50]. Propionic acid, among other short-chain fatty acids (SCFA), contributes to the health of colonocytes and has been shown to be depleted in colorectal cancer [51]. The discovery that subjects with colorectal cancer harbor less *Cutibacterium acnes* could potentially explain the previous reports of depletion of propionic acid in this population and may shed some light on the pathophysiology of disease development (Additional file 4: Table S3).

We acknowledge that there are limitations in our pipeline. One potential issue of MicroPro is under the situation that the core genomes of some microbes are present in the reference database while their corresponding pan-genomes are not; MicroPro will report the core genome in the known abundance profile and the remaining parts as separate unknown MAGs. This issue may not be problematic for the prediction of a disease using random forest as it can use one of the abundance profiles for phenotype prediction. However, caution is needed when the objective is to identify the microbes significantly associated with the disease since both the core genome and the corresponding MAG could be reported as associations although they are actually from the same genome.

We also acknowledge that although unknown features are extracted through assembly and binning, more functional analysis is needed to further understand the roles of each bin in diseases. Additionally, the disease prediction study is only observational and does not show the causality between a certain or a group of microbes and diseases. Furthermore, though we only tested MicroPro in disease-related analysis, MicroPro is ready to be applied to any type of phenotype prediction metagenomic studies. By fully utilizing both known and unknown organisms including viruses in the microbiota, we expect MicroPro will help to largely improve the prediction accuracy and facilitate biomarker detections.

## Conclusions

MicroPro provides a highly useful tool to study the associations between microbiota and diseases without neglecting key information from unknown organisms. The microbial prediction of disease can be useful in understanding disease pathogenesis and may become crucial in laying groundwork for future development of specific disease biomarkers.

## Methods

### Datasets

We downloaded all the datasets using the links provided in the original papers [8–11]. The number of cases and controls is given in Table 1. For Zeller_CRC, the “small adenoma” samples were treated as controls while the “large adenoma” samples were removed.

### MicroPro: a pipeline of predicting phenotypes based on metagenomic data

#### Step 1: Reference-based known microbial abundance characterization

We used Centrifuge [19] to map the reads to the microbial genomes and calculated the abundance profiles of known microbial organisms from the metagenomic data. In terms of Centrifuge command, we set flag “-q” which indicated the input was in fastq format and the other arguments were set as default. Centrifuge is an alignment-based taxonomic profiling tool. Its microbial database contains all the available bacterial, viral, and archaeal complete reference genomes in NCBI (up to January 4, 2018). Centrifuge also utilizes an expectation-maximization (EM) algorithm to compute the abundance for each microbial species. This EM-based algorithm is similar in spirit as those used in Cufflinks [52], Sailfish [53], and GRAMMy [54]. It takes into account reads mapped to multiple genomes or multiple locations in the same genome. In our study, we adopted the species abundance calculated by Centrifuge as the known microbial feature.

#### Step 2: Estimating abundance profiles of unknown microbial organisms based on reads assembly followed by contig binning

Although Centrifuge accurately characterizes known microbial relative abundance profiles, a large fraction of reads cannot be mapped to the known microbial organisms. The average mapping rate for each dataset is about 35–40% in our study (Additional file 1: Figure S3). The large amount of unmapped reads can potentially provide extra information on the prediction accuracy of phenotypes based on the metagenomic data. Therefore, our main objective in this step is to take into account the unmapped reads for phenotype prediction.

After filtering out mapped reads from the metagenomic data, we performed cross-assembly on the unmapped reads from all samples. We tested two assemblers: Megahit [33] and Minia 3 [27] in this step. Megahit assembles large and complex metagenomic data de novo based on succinct de Bruijin graph. Minia 3 utilized a more space-efficient bloom filter to perform sequence assembly. As shown in the “Results” section, Megahit performed better in real data analysis in terms of prediction but required much more computing time and memory than Minia 3. After cross-assembly, we used MetaBAT 2.12.1 [55] to perform binning on the assembled contig set. MetaBAT 2.12.1 is a reference-free metagenomic binner, and its binning criterion is based on tetranucleotide frequency and mean base coverage. This “reference-free” feature is crucial to our study, since the contig set to be binned contained no reads that could be mapped to a known reference. Recent comparative studies on contig binning [56] showed that MetaBAT 2.12.1 performs well compared to other contig binning algorithms.

Reads assembly and contig binning are highly important to recover unknown organisms from the unmapped reads. Here, “unknown organisms” represent the organisms without a known reference. Once we finished cross-assembly and metagenomic binning, we treated each contig bin as an unknown organism and the binned reads as a part of its genome. In terms of defining the feature of the unknown organisms, we still used the relative abundance, just as what we did for known species. The formula of the relative abundance (Ab) of unknown organism *i* was:

$$ \mathrm{Ab}(i)=\frac{rc_i}{\sum \limits_{j=1}^N{\mathrm{rc}}_j}, $$

where rc was the length normalized read counts, which was defined as the number of reads mapped to that organism divided by its genome length. Here, calculating rc was a major issue, since we do not know the whole genome of the unknown organism. To overcome this challenge, we first mapped all the unmapped reads back to the contig set using BWA-aln [57] with parameter “-n” set as 0.03 (only alignments with more than 97% accuracy were considered mapped). Then, we calculated the length normalized read counts (rc) for each contig according to the mapping results. Finally, for each contig bin (i.e., each unknown organism), we took the average rc of all the contigs that belonged to it as an approximation of its real rc. We could compute the unknown feature for all contig bins using the above formula. In terms of combining the known and unknown abundances, we calculated the mapping rate *α* (defined as the number of mapped reads/the number of total reads) for each sample and multiplied the known and unknown abundances by *α* and 1 − *α*, respectively, so that the combined abundance table sums to one for each sample.

#### Step 3: Predicting phenotypes using random forests

In the above two steps, we extracted the relative abundance profiles of both known and unknown microbial organisms. We then trained a random forests [23] classification model based on the combined abundance profiles to differentiate between the cases and the controls. Random forests is an ensemble of the decision tree algorithm and is highly robust to over-fitting when the number of features is greater than the number of samples. Our analysis was performed with R package “randomForest.” We randomly separated the dataset into training set and test set with a ratio of 7:3. During model training, we used tenfold cross-validation to tune the number of variables selected at each split, which is the “mtry” argument of the randomForest function in R, for best predictive performance. In terms of the measure of prediction accuracy, we adopted the area under the receiver operating characteristic curve (AUC) score, a widely used performance measure of the classification model. An AUC score close to 1 indicated perfect classification, while a 0.5 AUC score revealed that the model was close to a random guess. The above procedure was repeated 30 times.

### Reference-based and de novo assembly-based methods

Reference-based methods use a reference database to characterize microbial abundances. In this paper, the AUC scores for the reference-based method were obtained by training a random forest classification model based only on the Centrifuge abundance output (i.e., the known abundance table in the MicroPro pipeline). De novo assembly-based methods generate metagenomic assembled groups by assembly and binning of raw reads without the help of any reference genomes. To compare its predictive performance with MicroPro, we implemented de novo assembly-based method on all the four metagenomic datasets. We first generated a cross-assembly of all the metagenomic reads in a dataset. Due to insufficient computing memory, cross-assembling all samples using Megahit was computationally infeasible. Thus, we only used Minia 3 for cross-assembly. After obtaining the assembled contigs, we performed metagenomic binning of the assembled contigs by MetaBAT 2.12.1 and computed the contig bin abundances in the same way as the MicroPro pipeline. The abundance profile of bins was used as features for the random forest classification studies

### Simulation studies

We performed simulation studies to compare the predictive performance of MicroPro, reference-based method, and de novo assembly-based method. We simulated 50 shotgun metagenomic sequenced samples with 25 cases and 25 controls in the following way. To mimic the real human gut microbial community, the abundance profiles used in the simulation were modified based on the known abundance table of the QinN_LC dataset. In particular, we calculated the average relative abundance of the microbes at the genus level among all control samples and only kept the top 100 bacterial genera by the descending order of abundance. Then, we divided this abundance vector by its sum and treated it as the standard abundance profile of the control samples. For the case samples, we randomly selected 10 microbes and multiplied their abundances by *f*_*i*_, *i* = 1, …, 10, where each *f*_*i*_ was sampled from Uniform (0.1, 3). We renormalized the derived abundance vector to sum to 1 and used it as the standard abundance profile of the case samples. We also introduced absolute random Gaussian noise with mean zero and standard deviation equal to each component to the standard abundance profiles to further diversify the microbial composition of the simulated samples. CAMISIM [58] was then used to generate 50 samples with Illumina 2 × 150 bp paired-end reads based on the generated abundance profiles. Each generated sample had a size of 1 GB (500 Mbp).

MicroPro with different assemblers Megahit and Minia 3 was tested on the simulated datasets. Reference-based method only used the Centrifuge abundance output as the feature of the classification study. For this simulated dataset, we randomly picked 30 microbes out of 100 to generate the reference genome database used in Centrifuge taxonomic profiling. De novo assembly-based method generated metagenomic assembled groups by assembly and binning of raw reads without any reference genomes. We also tested two assemblers Megahit and Minia 3 for the de novo assembly-based method. The random forest classification analysis was performed in the same manner as step 3 in the MicroPro pipeline. Since we used predetermined abundance profiles to simulate metagenomic reads, we obtained the ground truth AUCs with these abundance profiles input as the classification feature.

### Predicting phenotypes based on virus abundance profiles

Viruses play a very important role in the human microbial community by controlling the balance of different bacterial organisms. However, due to its relatively low abundance, extraction of all the viral information, especially those without a known reference, remains a major difficulty. Aimed at making full use of all the viral features within metagenomic samples, the virus version of MicroPro is similar in spirit to the general pipeline presented in the previous section, except for an additional step for viral contig detection. The full pipeline is shown below.

#### Step 1: Known viral abundance extraction

For the known viral abundance, we again used the software Centrifuge, but only extracted the viral abundances from the Centrifuge profiling output and treated it as the known viral feature.

#### Step 2: Unknown viral feature detection

We performed cross-assembly using Megahit on the unmapped reads filtered out by Centrifuge results. Before metagenomic binning, we applied VirFinder [26] for viral contigs detection. VirFinder utilized a logistic regression model to differentiate between bacterial and viral contigs. We considered a contig as a virus if its VirFinder *q* value is smaller than 0.2. *q* value [59] is a *p* value correction method targeting exact false discovery rate (FDR) control. We performed metagenomic binning on the viral contigs and calculated viral bins’ abundance using the same method as described in the previous section step 2.

#### Step 3: Predicting phenotypes based on viral abundance

With both the known and unknown viral features at hand, the next step was to perform the prediction analysis. We combined two viral features in the same way as in the general MicroPro pipeline and trained a random forest model based on the extracted viral abundance. We used tenfold cross-validation to tune the parameters and set AUC score as the measure of prediction accuracy.

### Alpha diversity analysis

Alpha diversity is a widely used diversity measure in microbiome studies. It is defined based on both the number of species within a sample and the abundance of each species. We performed alpha diversity analysis of both microbial and viral abundance profiles. Alpha diversity with Shannon index is calculated by package “vegan” in R.

### Significantly associated microbial organisms for each disease

We identified the significantly associated features by the Boruta feature selection method [35]. Boruta is an iterative algorithm to select all relevant features through statistical tests. The analysis was carried out with R package “Boruta.”

### Predictive study between the two T2D datasets

We trained a random forest model based on one of the T2D datasets and tested it on the other to obtain the AUC score. Features included were also the known and unknown microbial abundance. Obtaining the known feature was essentially the same procedure as MicroPro’s step 1. We used the following strategy to calculate the abundance profiles of the unknown microbial organisms. For the train set, we used MicroPro’s step 2 with assembler Megahit to find out the unknown microbial feature. For the test set, instead of mapping back to its own contig set, we aligned the unmapped reads in the test set against the train data contig set. In this way, we could obtain a consistent feature matrix so that the following prediction analysis could be performed seamlessly.

## Additional files

Additional file 1 : Figure S1. Cumulative probability of alpha diversity of known profile. Plot A uses all the microbial abundances while plot B only uses viral abundances. For both plots, only known abundances are used for the calculation. Shannon index is set as the diversity index. WMW test *p* values between the cases and the controls are provided. Figure S2. Cumulative probability of alpha diversity of unknown profile. Plot A uses all the microbial abundances while plot B only uses viral abundances. For both plots, only unknown abundances are used for the calculation. Shannon index is set as the diversity index. WMW test *p* values between the cases and the controls are provided. Figure S3. Histograms of mapping rates of each dataset. Dashed lines show the mean mapping rate. (PDF 656 kb)

Additional file 2 : Table S1. Computing resources required for each method on four real metagenomic datasets are provided. MaxRSS refers to the maximum memory used by the corresponding method. (XLSX 10 kb)

Additional file 3 : Table S2. Number of assembled contigs from unmapped reads and number of detected viral contigs by VirFinder for each dataset are provided. (XLSX 9 kb)

Additional file 4 : Table S3. Significantly associated microbes selected by Boruta feature selection method for each dataset are provided. The table also shows the mean abundance in cases and controls for each selected microbes together with a FDR-adjusted WMW test *p* value for differences in the mean abundances. (XLSX 19 kb)

Additional file 5 : Table S4. A genus-level assignment for each MAG generated from four metagenomic datasets is given. The corresponding Mash distance and *p* value are also provided. A MAG with multiple hits in the database is reported in the table only if all of its hits belong to a common microbial genus in the taxonomy tree. (XLSX 21 kb)

## Data Availability

All the datasets used in this study are publicly available from the European Nucleotide Archive (ENA) database (https://www.ebi.ac.uk/ena). Accession number for ZellerG_CRC is ERP005534 [8], for KarlssonFH_T2D is ERP002469 [9], for QinN_LC is ERP005860 [11], and for QinJ_T2D is SRA045646 [10]. MicroPro is freely available at https://github.com/zifanzhu/MicroPro [60] and 10.5281/zenodo.3336360 [61] under the GNU General Public License (GPL), version 3. The simulated data used in this study is available at Zenodo [62].
