# Correction to: MicroPro: using metagenomic unmapped reads to provide insights into human microbiota and disease associations

**DOI:** 10.1186/s13059-019-1826-9

**Published:** 2019-10-22

**Authors:** Zifan Zhu, Jie Ren, Sonia Michail, Fengzhu Sun

**Affiliations:** 1 Quantitative and Computational Biology Program, Department of Biological Sciences, University of Southern California, Los Angeles, CA USA; 2 Department of Pediatrics, Division of Gastroenterology, Keck School of Medicine, University of Southern California, Los Angeles, CA USA

**Correction to: Genome Biol**

**https://doi.org/10.1186/s13059-019-1773-5**

Following publication of the original paper [1], Dr. Nayfach kindly pointed out an error and the authors would like to report the following correction.

On page 7, paragraph 3, line 13, the statement “Nayfach et al. suggested Mash distance of 0.35 as a genus-level threshold for microbes” is incorrect. Nayfach et al. [2] used phylogenetic distance instead of Mash distance to define genus and higher levels of taxonomy.

In order to determine the genus level threshold for Mash distance, we used Mash v.2.0 with sketch size 1,000,000 and k-mer size of 21 to calculate pairwise Mash distances between all the 11,444 complete bacterial genomes in the Centrifuge database (up to December 10, 2018). We then obtained the distributions of the pairwise Mash distances for three different groups of genome pairs: A. both are from the same species; B. the two bacterial genomes are from different species but the same genus; and C. the two bacterial genomes are from different genera. We could observe clear separations between the three groups of genome pairs under cutoff of 0.05 and 0.34 (Fig. 7). We found that 98.02% of group A distances are below 0.05, 92.37% of group B distances are above 0.05 and below 0.34, and 91.27% of group C distances are above 0.34. These results demonstrate that Mash distance thresholds of 0.05 and 0.34 can be reasonably used as species and genus level thresholds, respectively.

**Fig. 7:**
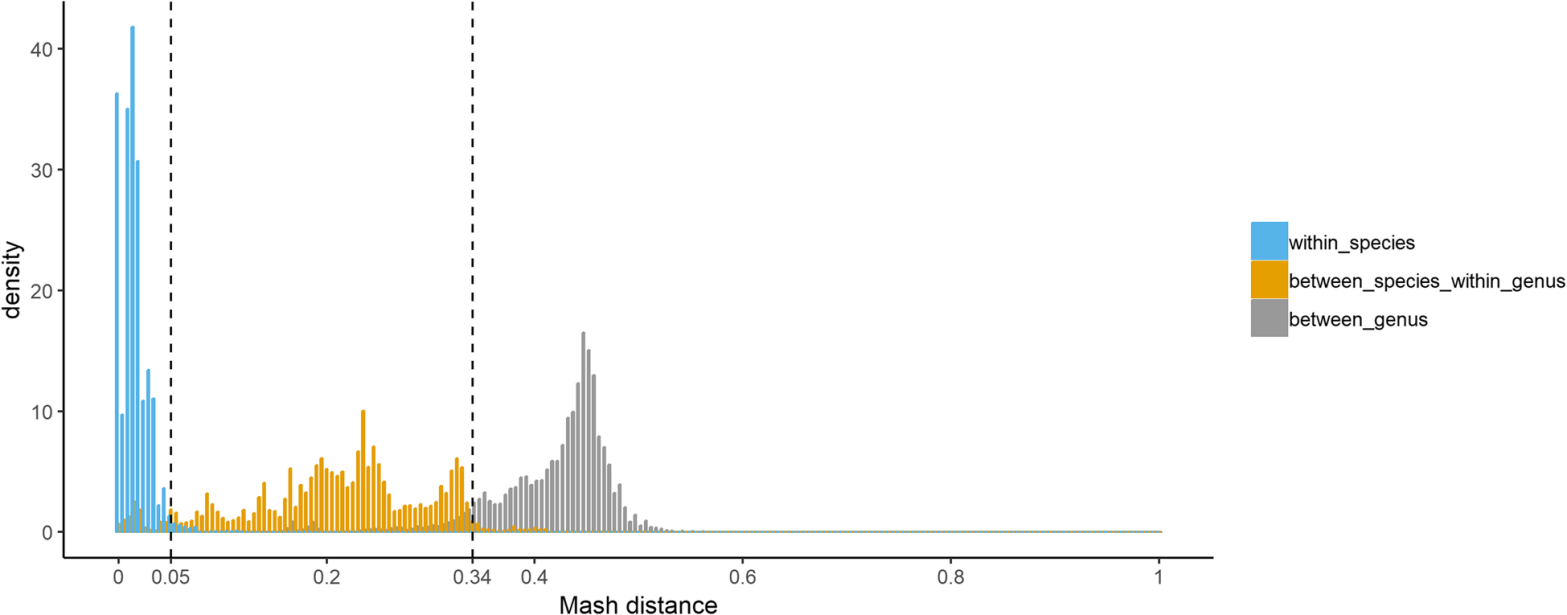
Histograms of pairwise Mash distances for all the complete bacteria genomes in the Centrifuge database. “within_species”, “between_species_within_genus”, and “between_genus” represent three different groups of genome pairs depending on whether the two bacteria genomes are: A. from the same species; B. from different species but the same genus; and C. from different genera. Mash v.2.0 with sketch size 1,000,000 and k-mer size of 21 is used to calculate the pairwise distances

In the original paper [1], we used Mash v.2.0 with default parameters in the taxonomic assignments of the MAGs generated in four datasets. However, default setting of Mash uses sketch size 1,000 which causes large variance in the calculated Mash distance [3]. In this correction, we redid the taxonomic assignments using Mash v.2.0 with sketch size 1,000,000. Under Mash distance threshold of 0.05 (species) and 0.34 (genus), we found that 0.65 and 19.83% of the total MAGs could be assigned to the species and genus levels, respectively. Detailed taxonomic assignment results are provided in the updated Additional file 5: Table S4.

## Supplementary information

**Additional file 5: Table S4.** Taxonomic assignments for each MAG generated from four metagenomic datasets. The corresponding Mash distance, number of matched sketches and the NCBI accession of the best hit are also provided. A MAG with multiple hits in the database is reported in the table only if all of its hits belong to a common microbial species/genus in the taxonomy tree.
