# Supplementary material for: MicroPro: using metagenomic unmapped reads to provide insights into human microbiota and disease associations
Source: Genome Biol. 2019 Aug 6;20:154. doi: 10.1186/s13059-019-1773-5 (PMC6683435; doi:10.1186/s13059-019-1773-5)
Supplement: Supplementary file 1 — : Figure S1. Cumulative probability of alpha diversity of known profile. Plot A uses all the microbial abundances while plot B only uses viral abundances. For both plots, only known abundances are used for the calculation. Shannon index is set as the diversity index. WMW test p values between the cases and the controls are provided. Figure S2. Cumulative probability of alpha diversity of unknown profile. Plot A uses all the microbial abundances while plot B only uses viral abundances. For both plots, only unknown abundances are used for the calculation. Shannon index is set as the diversity index. WMW test p values between the cases and the controls are provided. Figure S3. Histograms of mapping rates of each dataset. Dashed lines show the mean mapping rate. (PDF 656 kb) [file 13059_2019_1773_MOESM1_ESM.pdf]

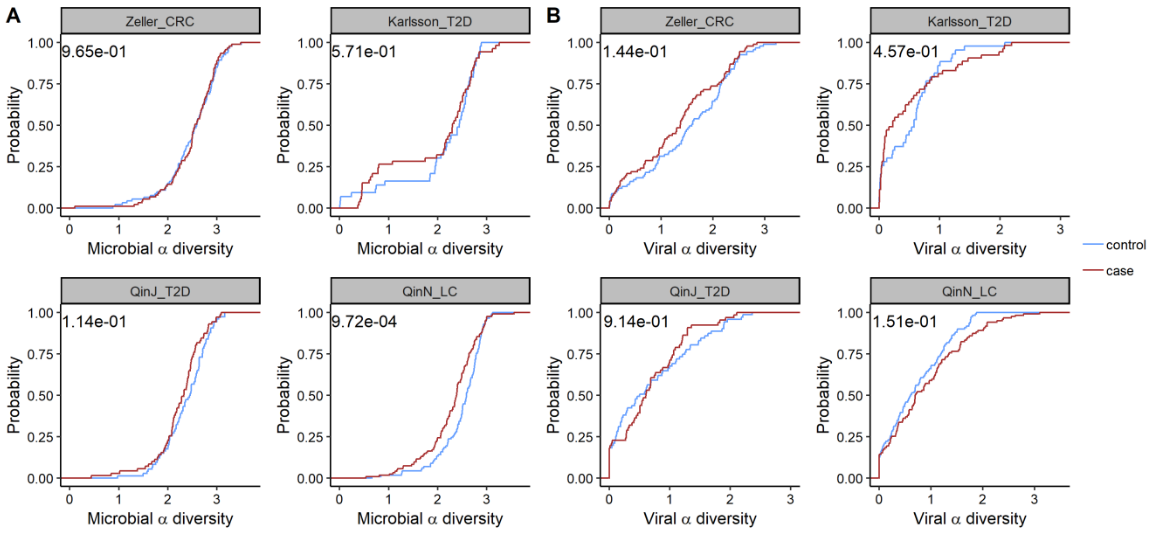

Fig. S1. Cumulative probability of alpha diversity of known profile. Plot A uses all the microbial abundances while Plot B only uses viral abundances. For both plots, only known abundances are used for the calculation. Shannon index is set as the diversity index. WMW test p-values between the cases and the controls are provided.

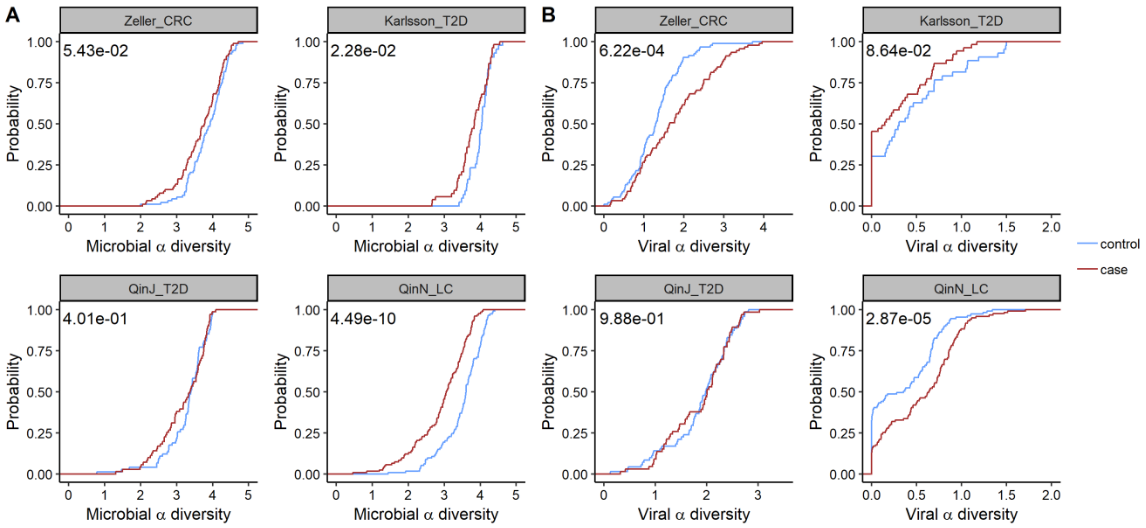

Fig. S2. Cumulative probability of alpha diversity of unknown profile. Plot A uses all the microbial abundances while Plot B only uses viral abundances. For both plots, only unknown abundances are used for the calculation. Shannon index is set as the diversity index. WMW test p-values between the cases and the controls are provided.

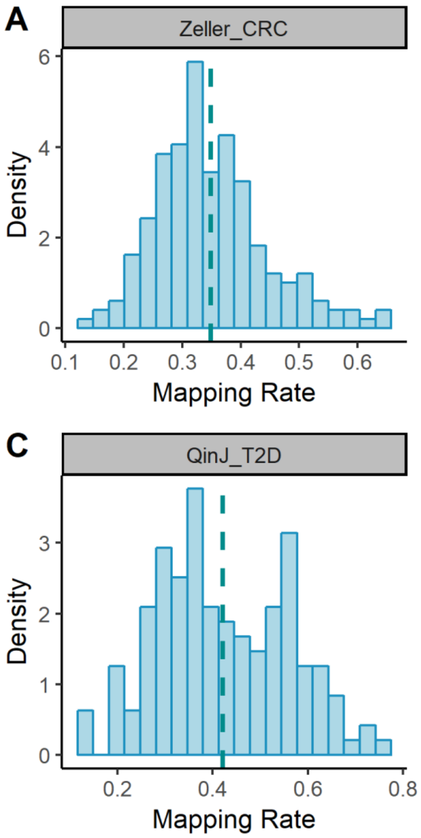

Fig. S3. Histograms of mapping rates of each dataset. Dashed lines show the mean mapping rate.
